# Practical Implications of the Organizational Commitment Model in Healthcare: The Case of Nurses

**DOI:** 10.1155/2024/6455398

**Published:** 2024-05-07

**Authors:** Mercedes Rodríguez-Fernández, Juan Herrera, Carlos de las Heras-Rosas, Antonio Manuel Ciruela-Lorenzo

**Affiliations:** ^1^Faculty of Social and Work Studies, University of Málaga, Málaga, Spain; ^2^School of Industrial Engineering, University of Málaga, Málaga, Spain

## Abstract

**Background:**

In addition to the usual difficulty of managing human capital in any organization, healthcare institutions have other problems to solve arising from the circumstances and the very nature of the work they perform, such as the ethical pressure on staff, emotional exhaustion, the distribution of work shifts, or the general shortage for nurses. In many cases, this situation has an impact on the quality of care.

**Objective:**

The main objective of this research is to compile, in a single document, human resource practices that help health centre managers improve results in terms of performance and quality of care, as well as avoid the intention of abandoning the job, specifically related to the work of nurses.

**Methods:**

To this end, a systematic literature review has been performed based on 229 papers published in the Web of Science database, from which the practical implications for nurses proposed by these authors have been extracted.

**Results:**

The main results suggest that developing affective commitment helps to improve organizational performance and enhance patient safety culture. Furthermore, improving communication and meaningfulness of work, recognition by superiors, or job flexibility would improve the quality of outcomes, for the work of nurses. *Conclusions and Implications for Nursing*. Stimulating normative commitment, reducing excessive control, and paying attention to job burnout and job stress help combat the intention of voluntary turnover or leaving the job, especially in the case of nurses.

## 1. Introduction

Healthcare faces a considerable challenge regarding the management of human resources [1, 2]. The World Health Organization (WHO) has already remarked, in the WHO's World Health Report [3], the worrying shortage of staff and management difficulties in healthcare. More recently, the Sustainable Development Goals Report of the 2030 Agenda [4] warns about the lack and unequal distribution of staff in health institutions.

Human resource management (HRM) in the healthcare sector has become an arduous task for the managers of these institutions, as in addition to the usual problems that can arise in any organization, there are others derived from the type of work that is carried out [5, 6], such as shift work, care pressure, emotional exhaustion, and work ethic. In any organization, it is essential that employees are aligned with the organizational goals, and in healthcare institutions, this aspect is even more important, as it is the healthcare professionals who are in direct contact with patients, and their involvement, empathy, and motivation will largely determine the quality of the medical care [7–9].

The singular characteristics of this sector have generated a great deal of research related to healthcare organizations [10, 11]. Amongst the approaches mainly employed, two main categories can be distinguished. On the one hand, the research describes specific cases of HRM in healthcare organizations in a particular location and subject to particular circumstances: Asian nurses in US hospitals [12], nurses in US hospitals [13, 14], Dutch cases [15, 16], Canadian staff nurses [17], and China's circumstances [18]. On the other hand, there is an investigation that addresses the different factors considered in the conceptual model underlying this research [19], such as human resource management [9, 20], job satisfaction [21, 22], organizational commitment [23–25], staff turnover [12, 26], risk of burnout [27, 28], or insufficient availability of qualified nursing staff [10, 13, 29, 30].

Published research has described and developed these issues, linking them to different organizational factors such as managers' leadership styles [31, 32] or psychological contract fulfilment [33, 34]. The latter's link to organizational and professional commitment has also been analysed [25, 35], and other concepts such as trust, performance, or productivity emerge from it. In fact, organizational commitment (OC) is positioned as one of the most important elements in the management of human resources in healthcare [23, 36].

As can be seen, there are many factors and interrelationships in the management of human resources in healthcare institutions that are also present in other organizations, perhaps taking on greater importance in these due to their nature and circumstances.

Therefore, there appears to be a high level of interest in the subject and a significant production of literature; however, as far as the authors are aware, the analysis of OC in HRM in the health sector has never been addressed from a practical perspective. In this sense, the aim of this research is to bring together in a single document the practical implications resulting from the different techniques and dynamics of HRM in healthcare institutions in which OC plays an essential role. This research will help all managers understand what factors moderate the OC of their employees (particularly nurses) and how they affect the performance and quality objectives in their organizations. It also presents causes and possible solutions to avoid the negative consequences of low OC in institutions. We also believe that this publication will provide the scientific community with interesting insights into OC in healthcare institutions and with reference to the practical implications to be adopted, which can be a starting point for future research.

## 2. The Review

Organizational commitment (OC) can be described in terms of the personal benefit of the functions performed, the autonomy to perform the job tasks, and the strategic management assumed by the employees [37]. In particular, according to Fadillaha et al. [38], OC describes employees' willingness to accept organizational goals and to cope with work. In this way, it encompasses a series of behaviours that lead them to make efforts for the good of the institution, to accept its values, and to long for permanence in the institution [39]. In this sense, Sena [40] goes a step further and incorporates aspects such as the desire to work well for the organization and have the pride of belonging to it.

Based on the above, and despite the existence of contrasting theories on OC [41–48], it can be observed that most authors agree with the three components proposed by Meyer and Allen [49, 50], i.e., continuance commitment, affective commitment, and normative commitment. These postulates are positioned as the starting point of this research; it is considered that OC is a key element in the achievement of objectives by organizations. This approach motivated us to explore the practical implications of achieving OC in healthcare organizations. Continuance commitment is based on the embeddedness caused by small investments that have developed over time [51]. Affective commitment is linked to psychological rewards such as recognition or support shown by peers that lead to identification with the organization and acceptance of its values and goals [52]. Finally, normative commitment relates to the worker's own values and his or her responsibility for his or her workplace ethics [51]. In this sense, Top et al. [53] highlight the importance of affective and normative commitment in the development of organizational trust, also highlighting the need for further research in this area.

Ultimately, organizations must have committed members if they are to thrive or even survive [54], and to this end, HRM activities have the potential to influence an employee's level of OC and, consequently, their retention [55]. This is valid in all areas and fields of activity. In healthcare, continuance commitment is considered one of the critical aspects due to the high turnover of staff, finding several types of research that related HRM to OC [56, 57]. Thus, Mousa and Puhakka [58], in the Egyptian setting, delved into the relationship between responsible leadership and organizational inclusion, concluding that an environment of respect, equality, and fairness in the workplace contributes positively to the development of affective, normative, and continuance commitment of physicians. Conversely, Ramoo et al. [59], in the Malaysian setting, established that there is a direct relationship between age and continuance commitment of nurses, although it also depends on external factors such as labour market opportunities.

Affective commitment also has a clear particularity for healthcare as it is positively related to job satisfaction and trust in the organization [16]. In that sense, Shipton et al. [9], in the context of several Dutch hospitals, argued that by performing roles that evoke deeply held values, such as excellent patient care and concern for others, line managers can have a positive effect on staff attitudes.

Furthermore, normative commitment has also been extensively addressed in the healthcare literature, where it is related to various aspects such as demographics, gender, background, age, and type of institution. In this sense, Gambino [60] established that the strongest indicator of the intention to remain in the position of nurses is normative commitment, which is also reinforced in the function of the age of the workers; he therefore recommends promoting normative commitment in younger nurses.

**Figure 1:**
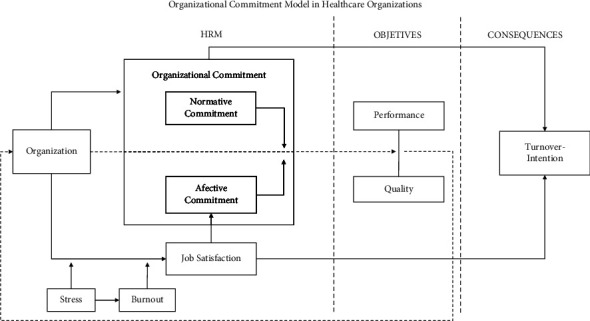
Model of organizational commitment. Source: Rodríguez-Fernández et al. [19].

Finally, also in relation to OC, Rodríguez-Fernández et al. [19] proposed a model that explained the effects of OC on healthcare institutions (Figure 1). They argued that, from a transformational leadership approach, OC becomes fundamental to achieve the high levels of performance and quality required in health-related workplaces. For this reason, they placed it in a central place amongst the aspects to be taken into account for the achievement of the objectives of these organizations. Another important aspect is job satisfaction, which is postulated as the main factor for improving OC, both normative and affective, and, in turn, avoids negative consequences such as the intention to leave the job. Similarly, certain moderating factors such as stress and burnout directly attack job satisfaction, negatively influencing it and thus OC, jeopardising the achievement of key objectives in healthcare institutions such as high performance and quality service to patients.

The model in Figure 1 provides an essential reference point to guide the process of this research. The systematic literature review undertaken represents a significant contribution to the study of OC in the context of healthcare organizations. This review brings together the range of practical implications detailed in previous research, comprehensively addressing all relevant factors that are usually dealt with individually. Other literature reviews on this topic have been developed previously. These include the longitudinal study by Freese et al. [33] on the impact of organizational changes on psychological contracts; the systematic review by Lu et al. [30] on job satisfaction of hospital nurses; the longitudinal study on OC and mental health [61]; or the systematic review on burnout syndrome in nurses [62]. No reviews have been previously found on the practical implications related to OC in healthcare institutions.

## 3. Methods

A systematic literature review of a sample of publications from the Web of Science (WoS) database has been carried out. To find a cause-effect relationship between OC and the objectives and consequences in healthcare organizations, the “model of organizational commitment” by Rodríguez-Fernández et al. [19] has been used.

### 3.1. Materials

**Figure 2:**
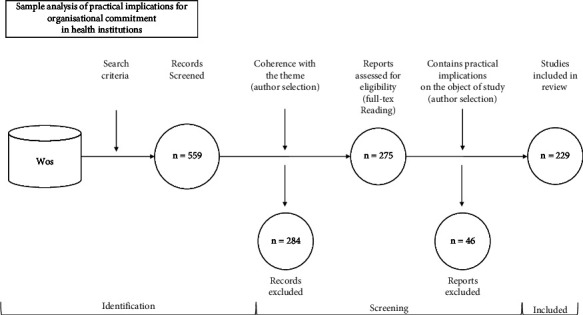
Sample analysis of practical implications for organizational commitment in health institutions and flowchart based on PRISMA 2020. Source: prepared by the authors. WoS (Web of Science).

The study has followed PRISMA 2020 guidelines for screening, selection, extraction, and data acquisition [63]. The search strategy is detailed in Figure 2, which explains the step-by-step process.

The documents analysed came from the Web of Science (WoS) database. The filters were established according to the criteria of the participants. The first restriction applied was the publication deadline, which was set to 31 December 2023. A preliminary scan was carried out to find the words that best represented the object of study. Publications containing these words in the title, abstract, author keywords, and keywords plus were filtered. For this, the following formula was used: (“organizational commitment” or “organizational commitment”) and (“health institutions” or “health system” or “healthcare” or “healthcare” or “health”). The search was restricted to the following indexes: Science Citation Index Expanded (SCI-EXPANDED), Social Science Citation Index (SSCI), Emerging Sources Citation Index (ESCI), Art and Humanities Citation Index (A&HCI), Conference Proceedings Citation Index-Social Science and Humanities (CPCI-SSH), Book Citation Index (BKCI), and Science Citation Index Expanded (CCR-EXPANDED). The year of publication was not limited, for the purpose of having the largest number of publications associated with this subject. To include the most recent publications that have not yet achieved the corresponding scientific impact, the number of citations received was unrestricted.

As a result of the search, a total of 559 manuscripts were obtained from WoS. After this first selection, the authors, individually, proceeded to discard those publications whose subject matter did not correspond to the purpose of the research or which, although of interest, did not provide practical implications related to OC in healthcare institutions. By using unique WoS, no duplicate papers were detected. A total of 275 documents were extracted from this first filter. Subsequently, the authors applied a second filter to extract those documents with practical implications, in line with the model used in this research—“model of organizational commitment” by Rodríguez-Fernández et al. [19], resulting in a sample of 229 documents (Figure 2). In the application of both filters, all authors agreed on the same number of excluded papers. Finally, the ATLAS.ti version 9 application was used for the literature review. This tool allows for the organization and categorization of large volumes of textual data, as well as, through the coding and development of the themes, the thematic analysis [64].

## 4. Results

The practical implications identified under each of model's headings are detailed as follows.

### 4.1. Organizational Commitment

In terms of works related to organizational commitment, 78 papers have been found. Amongst practical implications that have a general impact on OC, the following stand out: (a) when nurses in managerial positions are seen by their teams as effective and trusted transformational leaders, there is an improvement in their commitment to the organization, as well as an increase in worker effectiveness and productivity [65]; (b) the design and implementation of strategies and practices in HRM foster OC, which in turn improves well-being, happiness at work, and performance within the organization [66]; (c) in addition to the intrinsic needs of workers, the quality of the physical infrastructure and work environment are key factors in increasing OC [67]; and (d) nursing managers should take measures to reduce workload and improve remuneration and job autonomy to enhance OC [68].

Publications with practical implications related to affective commitment have highlighted the following issues: (a) increased affective commitment implies growth in job performance [69], and for the case of nonprofit organizations, it is also considered a key factor [70]; (b) increased affective commitment increases patient safety culture [71]; (c) it has been detected in organizations that high levels of affective commitment hinder the occurrence of job burnout [72]; and (d) employee training and development has a moderating effect on the growth of affective commitment [15].

With respect to normative commitment, the results suggest that (a) with regard to turnover intention, in the case of nursing jobs, improving job quality helps to avoid resignations [73]; (b) with regard to improving levels of *normative commitment*, and generating greater commitment, it is necessary to foster loyalty and obligation in healthcare settings and nursing schools [60]; and (c) in relation to the performance of healthcare staff, Gorgulu and Akilli [74] suggest that there is a positive and significant relationship between levels of normative commitment and job satisfaction displayed by workers, indicating how efforts made to reduce job burnout and psychologically supportive dynamics will improve motivation to provide better care.

It is noteworthy that another type of commitment analysed in the theoretical model, continuance commitment, has not been reflected in specific practices in the context of healthcare and no evidence of proposals or practical experiences has been found.

### 4.2. Moderating Factors: Job Satisfaction, Stress, and Burnout

The sample of publications presenting practical implications related to moderating factors such as job satisfaction, stress, and burnout consists of 45 papers. There has been a great proliferation of research focused on nurses. Suggestions [75] recommend that nurse executives support collegial solidarity in healthcare settings, in particular, by reducing stress, strengthening teamwork and communication, and thereby making the organizational climate more positive. In the study conducted by Tourigny et al. [76], moderating factors are highlighted for their practical importance for healthcare in general and for nurse management in the case of Japan and China in particular. Similarly, nurses' job satisfaction could be increased by promoting organizational and professional commitment and reducing occupational stress, role conflict, and role ambiguity [77]. In this sense, the authors in [78] adds, in the case of paediatric nurses, that, in addition to the above, it is necessary to provide support to alleviate turnover intention. Additionally, Perez [79] points out the significant cost of the loss of knowledge when employees decide to leave. In summary, burnout must be combated through interventions [80].

Furthermore, nurse managers should support burnout management with the provision of psychological employment [81] and practice responsible leadership by promoting a culture of inclusion [82]. Organizational culture should be geared towards prioritising workers' mental health, which requires improving communication skills and proactively engaging with teams to set and address wellness goals [61]. Burnout prevention plans, with a focus on social support, should be developed to improve nurses' quality of life and increase the care they provide [62]. Fragroso et al. [83] propose the reduction in work demands to prevent burnout, while Tripathy et al. [84] and Babatope et al. [85] suggest the enhancement of work-life balance practices, reduced burnout, and support from supervisors to increase job satisfaction. Management strategies that empower nurses for professional practice may be helpful in preventing workplace incivility and ultimately burnout [86]. Structural empowerment generates positive workplace outcomes; these outcomes relate to increased job satisfaction, increased OC, adoption of innovative behaviours, and reduced burnout and turnover [18, 87, 88]. Recently, Khatatbeh et al. [89] performed a systematic review and critical analysis of measures used to evaluate nurses' burnout and quality of life. Finally, applying resonant leadership in healthcare organizations can reduce employee burnout and prevent stress-related illnesses. It is also associated with a greater sense of belonging and OC [90].

### 4.3. Objectives: Performance and Quality

In the organizational commitment model that serves as the structure of this research, the organization's objectives focus on achieving high levels of performance and quality of care. A total of 35 papers have been analysed, which, in generic terms, try to apply HR practices that foster communication, work significance, and appreciation of superiors in order to increase OC and consequently the quality of services [91, 92].

In particular, it is recommended that managers and policy makers develop and implement supportive and nurturing strategies that improve organizational culture (emotional climate and collaborative relationships), which should lead in a reasonable time to more positive perceptions of the quality of healthcare [93, 94]. It is also suggested by Khera et al. [95] that health centres should measure performance in terms of the implementation of programmes that detect financial difficulties.

An imperative objective, according to Attia et al. [96], is to create a positive environment in intensive care units (ICUs) to increase staff satisfaction and efficiency by promoting quality of care, with special attention to junior staff.

Nurses also have a very important role in the analysed documents as a majority of authors consider them a stakeholder group on which management improvement should be focused. In particular, the proper establishment of work shifts is considered a measure of OC that would result in better performance of the nurses [97].

Nevertheless, managers should conduct professional training courses for nurses to improve the quality-of-service delivery [98]. In particular, the main implications for nursing education and practice are that educators and clinical mentors should work collaboratively to bridge the gap between theory and practice, thereby improving the quality of the clinical experience of these students, as proposed for China and elsewhere [99].

Along the same line, Hsu and Kernohan [100] recommend further research with different groups of nurses in a wider variety of work settings to examine the strengths and weaknesses of nurses and to develop appropriate strategies for the quality of their working lives. Research could seek to obtain objective measures of nurse performance rather than self-report measures [101].

### 4.4. Implications for Nursing and Health Policy

The consequences of the proposed model, considering its practical implications, are mainly based on improved performance, job satisfaction, and reduced turnover [102]. Some authors refer to structural empowerment leading to positive workplace outcomes such as increased satisfaction, greater commitment, adoption of innovative behaviours, and reduced burnout and turnover [87].

Organizations that need to adapt to changing environments should implement a strong employability culture, because such stimulates employability orientations amongst employees and, at the same time, decreases turnover intentions [103]. Monitoring and adopting measures that eradicate the experience of threats or violence by workers, as well as exposing physicians to less control over the pace of work, can prevent turnover intentions [104]. Workplace discrimination is associated with physicians' job turnover, career dissatisfaction, and contemplation of career change [105].

As regards nurses, it is important to pay attention to nurses' job burnout and job stress to improve job satisfaction and OC and to provide support to alleviate turnover intention [78]. Awareness of flexible work systems, OC, and quality of life needs to be reflected in interventions to reduce the turnover intention of health nurses [106]. It has been shown that healthcare organizations with greater investments in their nursing human capital are more likely to demonstrate lower levels of turnover of their registered nurses [107]. This has been corroborated by a recent mapping study on nurses' changing work practices performed by Salma and Waelli [108].

As for nurse leadership, authors such as Dahinten et al. [17], Alkarabsheh et al. [14], Lei et al. [109], or Orlowska and Laguna [110] assert that they should utilise a variety of organizational, structural, and psychological empowerment strategies that are important for their job satisfaction and potentially for the quality of patient care and turnover. Finally, as pointed out by Cooper-Thomas and Poutasi [111] and Rodríguez-Fernández [112], it is necessary to adequately adjust the person-position-organization triangle, which is key to avoid the intention to leave the organization.

## 5. Discussion

This study explores the influence of organizational commitment on performance and quality of care in healthcare settings, with a specific focus on nurses. It discusses the practical implications found in a systematic literature review, which suggests that effective OC can significantly improve both nurses' well-being and patient care outcomes.

The findings highlight that transformational leadership, effective communication, and recognition by superiors are essential to foster OC. Therefore, human resource management in the health sector becomes a challenging task for managers in these institutions. In these cases, employees must be aligned with the goals of the organization, as healthcare professionals will largely determine the quality of the healthcare provided [7–9].

Noteworthy, the present research demonstrates that there are numerous factors and interrelationships between human resource management and healthcare, something also reflected in previous studies [31–35].

In the organizational commitment model that serves as the structure for this research (Figure 1), the organization's objectives focus on achieving high levels of performance and quality of care, and OC is positioned as a key aspect, which is supported by research. These aims indicate that, in generic terms, HRM practices that promote communication are applied to increase CO and service quality [91, 92].

Numerous publications with practical implications related to affective commitment have been found to highlight its positive relationship with job performance [69] and an increase in patient safety culture [71]. This relationship becomes inverse in relation to occupational burnout [72]. However, employee training and development have a moderating effect on the growth of affective commitment [15].

Regarding normative commitment, and in line with the analysis conducted, there is a positive and significant relationship between levels of job satisfaction and the former [74]. This leads to improved job performance and low levels of turnover intention [73], so it seems advisable to foster loyalty and commitment in both healthcare settings and nursing schools [60, 113].

No evidence has been found in continuance commitment of proposals or practical experiences to promote this type of commitment. It should be noted that in the model on which this study is based [19], continuance commitment did not appear as representative, which confirms its correct approach.

The studies analysed indicate that transformational leadership, HRM practices, and both intrinsic and extrinsic factors have a positive impact on increasing CO. In particular, when nurses in managerial positions are perceived by their teams as effective and trusted transformational leaders, an increase in staff efficiency and productivity is observed, as well as an improvement in their commitment to the organization [65]. Furthermore, the implementation of HRM strategies and practices not only fosters OC but also improves well-being, happiness at work, and overall performance. Factors such as the intrinsic needs of workers, the quality of the physical infrastructure, and the work environment are key elements in increasing CO [67, 68].

Regarding job satisfaction, burnout, and stress as moderating factors, the results agree with Green et al. [80], who state that it is crucial to combat them with specific interventions. According to Tripathy et al. [84] and Babatope et al. [85], improvement of work-life balance practices and support from supervisors can increase job satisfaction. Velando-Soriano et al. [62] point out that burnout prevention plans with a focus on social support should be developed, which will improve nurses' quality of life and, consequently, the care they provide. Furthermore, the application of resonant leadership in healthcare organizations can decrease employee burnout and prevent stress-related illnesses [90].

Finally, the effective implementation of human resource practices aimed at promoting CO, and thus improving the quality of patient care and reducing the turnover rate of healthcare staff, shows significant variations between different healthcare settings. These differences are largely dependent, for example, on whether the health institution is publicly or privately owned, as well as on the geographical and cultural context in which it is located. In this regard, it is observed that nurses in the private sector and in rural areas more frequently consider leaving their posts compared with those working in the public sector and urban areas [66, 114]. Resource-constrained environments, however, create barriers that include shortages of skilled personnel, inadequate infrastructure, and limited access to continuous training [67, 115]. These barriers can negatively affect the implementation of HR practices aimed at fostering OC. In more developed contexts where resources are not a significant constraint, the challenges focus more on resistance to change and institutional bureaucracy [116]. In these environments, despite the availability of resources, rigid policies and lack of flexibility may impede the adoption of new HR practices that are essential to improve CO and, consequently, quality patient care. Therefore, to overcome these challenges, it is essential that HR interventions are designed and adapted considering the specific characteristics of each healthcare setting.

### 5.1. Practical Implications

The concept of organizational commitment amongst nurses has been studied in various hospitals, with a focus on its impact on healthcare quality and patient safety. In Mexico, a study on the dominant culture amongst nurses in a public hospital revealed the need for consensus on values, attitudes, and behaviours to strengthen and develop hospital organizations [117]. In Catalunya, Spain, a study on nursing governance and its impact on perceived quality showed a positive correlation between patient satisfaction and safety with the work of nurses and their commitment to the organization [118]. In the United States, research has explored the relationship between work engagement, burnout, and organizational commitment amongst nurses, with findings suggesting that job crafting and work engagement can mediate the relationship between participation in decision-making and intentions to leave the organization [119]. In Brazil, a study on leadership in intensive care units found that the relationship between nurses and doctors was more favourable in public hospitals with a more collaborative leadership style [120]. These studies highlight the importance of fostering a positive organizational culture and commitment amongst nurses to improve healthcare quality and patient safety.

### 5.2. Other Studies on Healthcare in Addition to Nursing

In addition to research on the organizational commitment of nurses in healthcare institutions, the literature has also addressed other healthcare-related work sectors, mainly physicians. Leadership behaviour has been shown to have a positive influence on the organizational commitment of physicians [121, 122], a finding that coincides with the results presented in this study for nurses. Research by Gokce et al. [123] related to the organizational commitment expressed by physicians in public and private healthcare providers is consistent with the results of our research, which show that there are a positive and significant relationship between perceived organizational support and affective and normative commitment and an insignificant relationship between perceived organizational support and continuance commitment.

However, there are also some differences in relation to the factors that enhance OC in doctors and nurses. Individual support from leaders and colleagues is positioned as elements that most positively influence the OC of physicians, while nurses improve their OC when they increase their degree of autonomy and do not feel overly monitored or overwhelmed at work [124, 125]. In summary, research shows that physicians have lower levels of CO than other health professionals, influenced by more work-related aspects, employment, and age [125].

### 5.3. Limitations and Future Research

As concerns the limitations of the present work and further research, we have delved into human resource practices in the health context, with a particular focus on nursing. However, it is necessary to recognise that our findings have inherent limitations. For instance, sector specificity may limit the generality of the findings to other contexts or populations. Furthermore, the use of additional databases could give as a result a larger number of papers. Furthermore, bibliographic bias could arise due to not using certain sources, Scopus, for example, which could lead to over-reliance on a particular perspective or ignoring relevant research that could challenge the assumptions of the study. However, the WoS database is highly valued in the scientific community due to its exhaustiveness and quality in indexing high-impact scientific journals in various disciplines.

In general terms, for future research it would be of interest to (1) study how the relationships between professionals and teams in healthcare impact key factors on organizational commitment and (2) apply the model used in this article to other healthcare groups for a holistic approach. Based on the results of this work referring to OC in future research, we will be able to follow up on new publications where nursing management is crucial in improving health. Regarding affective commitment, future research can be enriched with works that analyse the effects of its growth on patient safety, burnout occurrence, and the moderating effect of nurses' training and development. Regarding normative commitment, future research will analyse the relationship between nurses' job satisfaction and their improvement in daily motivation.

Finally, the inclusion of Magnet hospitals in the study of the applicability of this model can result in interest for an upcoming paper as these types of organizations present, amongst other benefits, better work environments, higher nurse job satisfaction, less burnout, and decreased intent to leave [126].

## 6. Conclusions

As noted above, this paper applies a previous theoretical model on organizational commitment in healthcare to the case of nurses.

The increase in OC is positioned as a key factor in achieving the objectives set by organizations, an effect that becomes even more transcendental in healthcare institutions. In the systematic review of the literature related to practical implications, the relevance of the high status of nurses, the importance of adequate human resource management, and the convenience of adequately covering the intrinsic and extrinsic needs of workers have been highlighted. Appropriate workload management, remuneration, and autonomy are also positioned as good practices to improve CO.

Organizational commitment can be divided into affective, normative, and continuance commitment. In the 229 articles analysed, there is little presence of practices related to improving continuance commitment. However, issues related to affective and normative commitment are sufficiently represented.

Affective commitment is the most present in the research and is positioned as key in nonprofit and healthcare organizations. It helps improve patient safety culture and decreases the occurrence of burnout, amongst other effects. Employee training and development have moderating effects on this commitment. It can be added that affective commitment implies an increase in job performance, specifically for the work of nurses.

To enhance normative commitment, loyalty and obligation need to be fostered in healthcare settings and schools. Job satisfaction favours normative commitment; hence, leaders should orient their human resource policies to reduce job burnout, encourage communication, increase flexible working hours, avoid excessive control, and, in short, generate a suitable environment for performance.

Job satisfaction, stress, and burnout are presented as moderating factors in achieving high levels of commitment. The authors highlight how these elements become more important in jobs related to healthcare in general and nursing work in particular, mainly due to the importance of the loss of knowledge when an employee decides to leave, and the negative influence that inadequate human resource management can have on patient care.

Health organizations aim to improve their performance levels and quality of care. HR practices for nurses that foster communication, job satisfaction, appreciation of superiors, and appropriate work environment will provide higher levels of CO, which will result in increased recognition of the work and improved patient care.

## Data Availability

This study has followed PRISMA 2020 guidelines for screening, selection, extraction, and data acquisition. The documents analysed came from the Web of Science (WoS) database.
